# Electroencephalographic Functional Connectivity With the Tacit Learning System Prosthetic Hand: A Case Series Using Motor Imagery

**DOI:** 10.3389/fnsyn.2020.00007

**Published:** 2020-02-28

**Authors:** Katsuyuki Iwatsuki, Minoru Hoshiyama, Shintaro Oyama, Hidemasa Yoneda, Shingo Shimoda, Hitoshi Hirata

**Affiliations:** ^1^Department of Hand Surgery, Graduate School of Medicine, Nagoya University, Nagoya, Japan; ^2^Department of Health Sciences, Faculty of Medicine, Nagoya University, Nagoya, Japan; ^3^Center of Brain Science (CBS), CBS–TOYOTA Collaboration Center, RIKEN, Nagoya, Japan

**Keywords:** tacit learning, electroencephalography, prosthesis, supplementary motor area, neuroplasticity, Hand20, brain function, neurorehabilitation

## Abstract

We previously created a prosthetic hand with a tacit learning system (TLS) that automatically supports the control of forearm pronosupination. This myoelectric prosthetic hand enables sensory feedback and flexible motor output, which allows users to move efficiently with minimal burden. In this study, we investigated whether electroencephalography can be used to analyze the influence of the auxiliary function of the TLS on brain function. Three male participants who had sustained below-elbow amputations and were myoelectric prosthesis users performed a series of physical movement trials with the TLS inactivated and activated. Trials were video recorded and a sequence of videos was prepared to represent each individual’s own use while the system was inactivated and activated. In a subsequent motor imagery phase during which electroencephalography (EEG) signals were collected, each participant was asked to watch both videos of themself while actively imagining the physical movement depicted. Differences in mean cortical current and amplitude envelope correlation (AEC) values between supplementary motor areas (SMA) and each vertex were calculated. For all participants, there were differences in the mean cortical current generated by the motor imagery tasks when the TLS inactivated and activated conditions were compared. The AEC values were higher during the movement imagery task with TLS activation, although their distribution on the cortex varied between the three individuals. In both S1 and other brain areas, AEC values increased in conditions with the TLS activated. Evidence from this case series indicates that, in addition to motor control, TLS may change sensory stimulus recognition.

## Introduction

Our movements are supported by conscious and unconscious motor control mechanisms (Steenbergen et al., 2010; D’Ostilio and Garraux, 2012; Hayashibe and Shimoda, 2014). Riding a bicycle, for example, requires an intricate interplay between the brain and muscles. A bicyclist must continuously adapt to widely ranging environmental changes while regulating balance, direction, and speed.

When we use an implement to extend the reach of our hands, the neural representation of our body is modified and the multisensory peripersonal space surrounding it is extended (Maravita and Iriki, 2004; Holmes, 2012; Juett and Kuipers, 2019). The tool is expeditiously integrated within our body schema. After upper limb amputation, the human control system generates neuromuscular adaptations to effectively utilize a tool such as a prosthetic. Compensatory mechanisms and sensorimotor integration are important for such adaptations (Claret et al., 2019). Wearing a prosthesis increases the perceived stump length and extends the peripersonal space boundaries so as to include it, such that the prosthesis partially replaces the missing limb (Canzoneri et al., 2013).

To reconstruct such natural motor control in robots, in addition to generating accurate motor output, implementing good sensory feedback is required in the internal processing system. However, the development of a technology that can perform these processes has been difficult to date because a feedback system with high precision is limited in its ability to flexibly respond to unanticipated environmental changes.

The Tacit Learning System (TLS; Shimoda and Kimura, 2010; Shimoda et al., 2013) is a closed-loop control system that allows adaptive movement in response to unexpected environmental changes. In its function, it contributes to a strategy of sensory feedback called sensory synergy (Alnajjar et al., 2015). The TLS is superior to conventional methods in terms of learning speed, cost, and robustness.

We integrated the TLS within a myoelectric prosthetic hand (Oyama et al., 2016) that automatically controls forearm pronosupination in accordance with the intention of the user. We demonstrated that this myoelectric prosthetic hand enabled sensory feedback and flexible motor output, allowing users to move naturally with little burden.

However, it is not easy to evaluate the internal and external benefits and costs involved in the use of a myoelectric prosthetic hand. A better motor output does not always correspond to less exertion. Therefore, we hypothesized that the effort to use a prosthetic hand, including the amount of neural activity required for motor control and sensory feedback, might be reflected in brain activity.

Imaging methods such as functional magnetic resonance imaging (fMRI) and near-infrared spectroscopy (NIRS) have been among the leading techniques for studying brain function in recent years. Both measure changes in blood flow within the brain; however, inherent disadvantages in the technologies, particularly in studies involving tool use, include the prohibition on metal materials in the former and low special resolution in the latter (Iwatsuki et al., 2020). The temporal resolutions of fMRI and NIRS extend from hundreds of milliseconds to seconds, insufficient to analyze human movement.

To overcome this problem, we employed magnetoencephalography during periods in which participants viewed previously recorded video of motor tasks, which is a feasible method for functional neuroimaging of the mirror neuron system (Wright and Jackson, 2007; Wang et al., 2008). In a preliminary study about the effect of TLS on brain function using magnetoencephalography, we observed stronger connections between the motor area and other cortical areas when the technology was used, as indicated by a significant increase in coherence values (Iwatsuki et al., 2019). Magnetoencephalography has high temporal resolution with reliable source estimation (Iwatsuki et al., 2019). It can safely measure the magnetic field generated by neuronal electrical activity. Therefore, highly accurate identification of source activities and connectivity measurements in regions of interest is possible (Iwatsuki et al., 2016). However, it is expensive and access is limited.

In contrast, electroencephalography (EEG) is readily available and can measure the same phenomena as magnetoencephalography based on the relationship between the magnetic field and electric current. Furthermore, recent advantages in source modeling and computing techniques have enabled us to estimate neural activity from EEG signals obtained with a limited number of electrodes. The technique of collecting EEG during video imaging has been established to evaluate hand function (Ikeda et al., 2019). Much is to be learned about how neural representations of the body are remodeled by amputations and the use of prosthetics. Therefore, in this study, we investigated the effect of the use of a TLS-equipped prosthetic hand on brain activity.

## Materials and Methods

The study protocol was approved by the Ethics Committee of the Nagoya University School of Medicine. Each volunteer gave informed consent prior to their involvement. Participants were three adult males, each with a below the elbow amputation who used a myoelectric prosthetic lower arm and handpiece in his daily life. Participant 1 had sustained a left arm amputation and participants 2 and 3 right arm amputations.

### Tacit Learning System (TLS)

The TLS-integrated prosthetic arm with a handpiece contains a closed-loop control system that supports user-initiated action resulting in functional motor synergy. It relies on the signal accumulation in its process of adaptation, during which a formative behavior is tuned into movement suiting a goal. The integrated algorithmic system is based on human kinesiology. It consists of three goniometric sensors (shoulder flexion, abduction and rotation) coupled with a handpiece equipped with hand grip and wrist rotation actuators. In this study, the system was attached to the participant’s own myoelectric prosthetic socket.

### Physical Movement Phase

Participants wore the prosthetic hand equipped with the TLS and were asked to perform a physical movement task with the system inactivated and then repeat it with the system activated. Three bars placed on a table in front of the seated participant were to be moved from a horizontal to a vertical position and then returned to the original horizontal position. This sequence was to be repeated three times, which was considered one trial. Finger manipulation to grasp and release each bar was performed by means of conventional electromyography electrodes in the arm socket, while forearm pronosupination was supported by the TLS. Everyone was able to complete the task with only simple instructions. Participants were asked to repeat the task, performing approximately 20 subsequent trials in 10 min, until an expected level of performance where an optimal balance of energy to effort was achieved.

All movements were videotaped, and a series of two recordings lasting 30 s each were made of a selected trial undertaken each participant, one with the TLS inactivated and one with the TLS activated. The usability of the prosthetic hand under both conditions from the perspective of each participant was evaluated by means of Hand20, an assessment of upper limb function (Suzuki et al., 2010; Iwatsuki et al., 2014).

### Motor Imagery Phase

In an EEG suite, each participant was seated directly in front of a large video monitor and asked to watch a sequence of the two previously recorded 30-s videos of himself while imagining performing the task under the condition presented. He was to actively perceive controlling the prosthesis in synchronization with what appeared on the monitor. The videos were presented six times, separated by a 20-s blank white interval screen.

### EEG Recording

EEG (Neurofax, Nihon Kohden Company, Tokyo, Japan) was taken during the motor imagery phase. EEG signals were recorded with 21 conventional passive gel electrodes based on the International 10-20 system. Continuous EEG signals were recorded using an initial bandpass filter between 3 and 120 Hz with a sampling frequency of 1,000 Hz. The impedance between electrodes was kept below 5 kΩ. Artifacts caused by heartbeat and ocular movements or blinks were rejected using electrocardiogram and Fp1 recording by signal space projection (Nolte and Hämäläinen, 2001).

### EEG Analysis

EEG signals were collected during the 30-s motor imagery phases (without and with TLS activated). To avoid any possible brain reaction at the onset of the video, EEG signals from the first 5 s were discarded, yielding 25 s series for analyses. Brainstorm software (Tadel et al., 2011) was used for all analyses. The cortical surface of a standardized brain base on MNI/ICBN152 (Fonov et al., 2009) as divided into matrixes with 3,001 vertices and electrical current in each vertex was estimated by minimum norm estimation (Baillet et al., 2001). First, the mean cortical electrical current during each task was estimated. Then, the bilateral supplementary motor area (SMA) was set as a region of interest, and neural connectivity between mean neural signals in the SMA and those in other vertices were calculated. The amplitude envelope correlation (AEC) of orthogonalized signal pairs for the alpha frequency band (8–12 Hz) was used to estimate neural connectivity (Colclough et al., 2016). Each AEC value was expressed as a correlation coefficient between –1 and 1. The mean signal in the left and right SMA was calculated because the separation was ambiguous. Differences in the mean cortical current and AEC values between the SMA and each vertex for the motor imagery tasks were calculated for each participant. AEC values of the alpha frequency band between the primary somatosensory areas (S1) for arms and hands, as distinguished from other brain areas, were also calculated during the motor imagery task with the TLS inactivated and activated.

## Results

The characteristics of the three participants are shown in Table 1. Compared to when the TLS was inactivated, all Hand20 scores of the TLS activated condition substantially improved.

**Table 1:** Participant characteristics.

Participant	1	2	3
Age (range)	45–49	50–54	55–59
Amputation side	left	right	right
Period after amputation (years)	6	18	6
Hand20 scores, TLS inactivated	47	31	69
Hand20 scores, TLS activated	43	22	61

**Figure 1:**
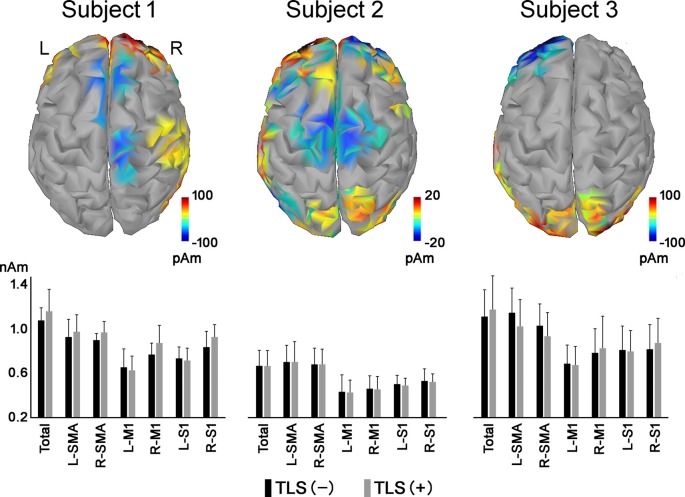
Differences in mean cortical current during motor imagery tasks without and with TLS activation [(movement imagery with TLS activation)—(movement imagery without TLS activation)]. Yellow to red and blue indicates increased and decreased current (pAm) during the task with and without TLS activation, respectively. Mean cortical current density in the total cortex (Total), SMA, M1, and S1 areas was shown in the bar graph below. Black and gray bars indicate cortical current density during movement imagery without and with TLS activation, respectively. Each vertical bar indicates a standard deviation. TLS, Tacit Learning System; SMA, supplementary motor area; M1, primary motor cortex; S1, primary somatosensory cortex.

Differences in mean cortical current between the motor imagery tasks without and with TLS activation varied among the three participants (Figure 1). Yellow to red and blue indicate increased and decreased current (pAm) without and with TLS activation. Figure 1 and Table 2 show inter-individual variation in cortical current density and AEC values during the imagery task. The variation suggests that the strategy in neural processes to control the prosthesis was different among participants. Reorganization of the function might be made in various ways as reported in a previous study (Belardinelli et al., 2017).

**Table 2:** Differences in mean AEC value between cortical areas during movement imagery tasks with and without tacit learning system (TLS) activation (movement imagery with TLS activation) − (movement imagery without TLS activation).

	Lt-SMA	Rt-SMA	Lt-Sensorimotor	Rt-Sensorimotor
**Participant 1**
Lt-Sensorimotor	−0.056	**0.027**	-	**0.034**
Rt-Sensorimotor	0.021	**0.045**	0.034	-
Lt-Frontal	0.217	**0.059**	−0.176	−**0.192**
Rt-Frontal	0.198	**0.071**	0.010	**0.061**
Lt-SMA	-	−**0.033**	−0.056	**0.021**
Rt-SMA	−0.033	-	0.027	**0.045**
**Participant 2**
Lt-Sensorimotor	−**0.185**	0.001	-	0.142
Rt-Sensorimotor	−**0.053**	0.067	**0.142**	-
Lt-Frontal	**0.113**	0.032	−**0.086**	−0.039
Rt-Frontal	**0.153**	0.105	−**0.109**	0.127
Lt-SMA	-	−0.021	−**0.185**	−0.053
Rt-SMA	−**0.021**	-	**0.001**	0.067
**Participant 3**	
Lt-Sensorimotor	**0.190**	0.119	-	0.019
Rt-Sensorimotor	**0.157**	0.063	**0.019**	-
Lt-Frontal	**0.189**	0.105	**0.120**	0.061
Rt-Frontal	**0.188**	0.034	**0.128**	0.012
Lt-SMA	-	0.208	**0.190**	0.157
Rt-SMA	**0.208**	-	**0.119**	0.063

*Positive (unshaded) values indicate increments of neural connectivity in AEC value. Columns with bold values indicate cortical areas contralateral to the hand amputated. AEC, amplitude envelope correlation*.

**Figure 2:**
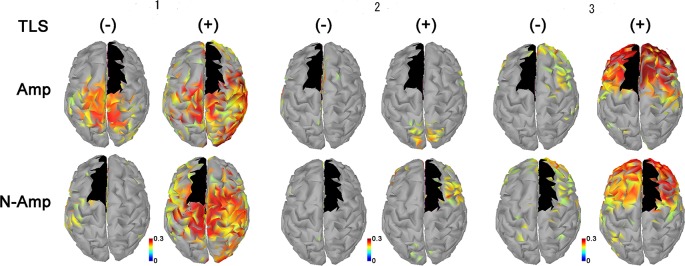
Neural connectivity expressed as amplitude envelope correlation (AEC) values for alpha frequency band between the SMA (black areas) and other brain areas without [TLS (−)] and with [TLS (+)] in the three participants. AEC values between SMA contralateral (upper row; Amp) and ipsilateral (lower row; N-amp) to the arm amputated and other areas are shown. All participants had increased AEC values in the condition with TLS activation between both SMA and other brain areas. Differences between Amp and N-amp sides were not obvious in participants. AEC, amplitude envelope correlation; SMA, supplementary motor areas; TLS, Tacit Learning System.

**Figure 3:**
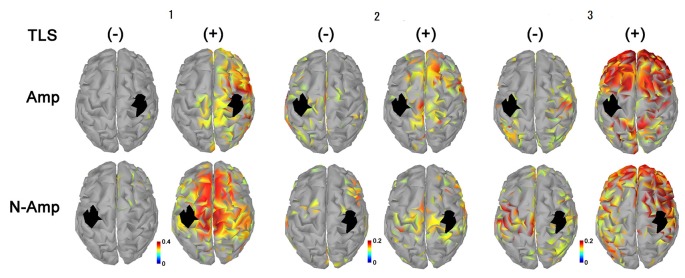
Neural connectivity expressed as AEC values for alpha frequency band between the primary sensorimotor area for arm and hand (S1, black areas) and other brain areas without [TLS (−)] and with [TLS (+)] in the three participants. AEC values between S1 contralateral (upper row; Amp) and ipsilateral (lower row; N-amp) to the arm amputated and other areas are shown. All participants had increased AEC values in the condition with TLS activation between both SI and other brain areas. Differences between Amp and N-amp sides were not obvious in participants. AEC, amplitude envelope correlation; TLS, Tacit Learning System.

All participants had increased AEC values in the condition with TLS activation between both SMA or S1 and other brain areas. Differences between Amp and N-amp sides were not obvious in any participant (Figures 2, 3).

## Discussion

A prosthetic system should ideally reinstate the bidirectional communication between the user’s brain and its end effector by restoring both motor and sensory functions lost after an amputation. Even without explicit feedback, grasping using a prosthesis partly relies on sensory information (Wilke et al., 2019). Furthermore, for smooth and efficient motor control, the brain needs to make fast corrections to resist possible perturbations, so both feedback corrections and feedforward adaptation are required (Yousif and Diedrichsen, 2012).

Functional connectivity may change in adaptation to the use of tools. Tools that easily induce brain adaptations and assist movements have a high user affinity. By means of motor imagery, in this study, we investigated changes in brain activity during the use of our newly developed prosthetic hand. These results are important to assess the prosthetic hand function and how the participants use their brains. Our results showed that the connectivity between the SMA and surrounding areas was increased by the support of the pronosupination movement with the TLS. This indicated that the SMA was associated with sequential movements, including arm and finger derived pronosupination and grasping.

In order to interact successfully with the environment, it is important to know precisely where our body parts are located in space and where the targets of our interactions are situated with respect to other people’s bodies and ours. Moreover, we need to maintain these topographical inputs updated as a function of ongoing and upcoming hand movements (Brozzoli et al., 2014). When we use tools, we alter our brain activity according to the type of tool to achieve adaptation. After achieving proficiency with a tool, the body image is expanded so that the user can perceive the tip of the tool as part of their body. This human adaptation is achieved by altering brain activity (Garbarini et al., 2015; D’Angelo et al., 2018).

In this study, we focused on activity in the SMA based on the findings of previous studies (Iwatsuki et al., 2019). The SMA plays a crucial role in domain-general sequence processes, contributing to the integration of sequential elements into higher-order representations regardless of the nature of such elements as motor, temporal, spatial, numerical, and linguistic (Kim et al., 2016). The SMA supports the sequence operations in a variety of cognitive domains that share an inherent sequence processing. These include action, time and spatial processing, numerical cognition, music and language processing, and working memory (Cona and Semenza, 2017). Furthermore, the SMA is involved in the planning and coordination of movement sequences (Schramm et al., 2018; Busan et al., 2019).

As shown in the movement imagery phase, neural connectivity between S1 for arms-hands and other brain areas increased during TSL activation. Increased neural connectivity was observed for S1 in both hemispheres. Although we could not refer to a specific neural activity changed by TLS, in terms of brain activity its effects were clearly observed. Participants 2 and 3 showed greater increases in neural connectivity between S1 contralateral to the arm amputated as compared to that ipsilateral and to other brain areas, but the laterality was not clear in participant 1. S1 receives information about motor output before the arrival of sensory feedback signals, suggesting that S1 executes the online processing of somatosensory signals *via* interactions with the anticipatory information (Umeda et al., 2019). This prosthetic system has the possibility to reinstate the bidirectional communication between the user’s brain and its end effector by restoring both motor and sensory functions lost after an amputation (Wilke et al., 2019).

This study had several limitations. It was conducted with only three participants. In addition, while following a method established elsewhere, the impact of TLS was ascertained solely from EEG results of the motor imagery phase. We attempted to record EEG during conditions with the TLS inactive and active for both the physical movement (bar relocation task) and motor imagery (video viewing) phases. However, during each condition in the physical movement phase, there was excessive motion noise. While EEG signals were collected continuously during the motor imagery phase, the two were not precisely synchronized. Last, we cannot show the differences in laterality. This could be attributed to the small sample size or because EEG does not have as high sensitivity and specificity as magnetoencephalography.

The increased AEC values observed during the motor imagery phase when the TLS was activated were similar to our previous results (Iwatsuki et al., 2019). But further, TLS use increased AEC values in both S1 and other brain areas. When we use tools, sensory-motor integration must occur. This is the process by which these two sensory and motor systems communicate and coordinate with each other. TLS may change not only motor control but also sensory stimulus recognition.

## Data Availability Statement

The datasets generated for this study are available on request to the corresponding author.

## Ethics Statement

The studies involving human participants were reviewed and approved by the Ethics Committee of the Nagoya University, School of Medicine. The patients/participants provided their written informed consent to participate in this study.

## Author Contributions

KI, SO, MH, SS, and HH performed the measurements. KI and MH evaluated the EEG results. KI, MH, and HY composed the manuscript. All authors discussed the results and contributed to the final manuscript.

## Conflict of Interest

The authors declare that the research was conducted in the absence of any commercial or financial relationships that could be construed as a potential conflict of interest.
